# Long-term trends in the prevalence of patients hospitalized with ischemic stroke from 1995 to 2010 in Sweden

**DOI:** 10.1371/journal.pone.0179658

**Published:** 2017-06-16

**Authors:** Kok Wai Giang, Zacharias Mandalenakis, Susanne Nielsen, Lena Björck, Georgios Lappas, Martin Adiels, Christina Jern, Annika Rosengren

**Affiliations:** 1 Department of Molecular and Clinical Medicine, Sahlgrenska Academy, University of Gothenburg, Gothenburg, Sweden; 2 Institute of Health and Care Sciences, Sahlgrenska Academy, University of Gothenburg, Gothenburg, Sweden; 3 Health Metrics Unit, Department of public health and community medicine, Sahlgrenska Academy, University of Gothenburg, Gothenburg, Sweden; 4 Institute of Biomedicine, Department of Clinical Pathology and Genetics, the Sahlgrenska Academy, University of Gothenburg, Gothenburg, Sweden; Stanford University School of Medicine, UNITED STATES

## Abstract

**Objective:**

The prevalence of stroke is expected to increase partly because of prolonged life expectancy in the general population. The objective of this study was to investigate trends in the prevalence of patients hospitalized with ischemic stroke (IS) in Sweden from 1995–2010.

**Methods:**

The Swedish inpatient and cause-specific death registries were used to estimate the absolute numbers and prevalence of patients who were hospitalized with and survived an IS from 1995–2010.

**Results:**

The overall number of IS increased from 129,418 in 1995 to 148,778 in 2010. In 1995, the prevalence of IS was 189 patients per 10,000 population. An increase in overall prevalence was observed until 2000, and then it remained stable, followed by a decline with an annual percentage change of (APC) -0.8% (95% CI -1.0 to 0.6) and with a final prevalence of 199 patients per 10,000 population in 2010. The prevalence of IS in people aged <45 years increased from 6.4 in 1995 to 7.6 patients per 10,000 population in 2010, with an APC of 2.1% (95% CI 0.9 to 3.4) from 1995–1998 and 0.7% (95% CI 0.6–0.9) from 1998–2010. Among those aged 45–54 years, the prevalence rose through the mid to late 1990s, followed by a slight decrease (APC: -0.7%, 95% CI-1.1 to -0.4) until 2006 and then remained stable with a prevalence of 43.8 patients per 10,000 population in 2010. Among ≥85 years, there was a minor decrease (APC: -0.3%, 95% CI -0.5 to -0.1) in overall prevalence after 2002 from 1481 to 1453 patients per 10,000 population in 2010.

**Conclusion:**

The overall prevalence of IS increased until 2000, but then remained stable followed by a slight decline. However, the prevalence of IS in the young increased through the study period. The absolute number of IS survivors has markedly increased, mainly because of demographic changes.

## Introduction

In 2015, stroke-related disease caused more than 6 million deaths worldwide and is considered as one of the most common causes of death and disability among adults [1,2] For the past 2 decades a significant decline in overall mortality and incidence of stroke have been observed in many West European countries [3–6]. In Sweden, the incidence of ischemic stroke (IS) has decreased among older people, but increased among younger adults (<45 years) [7]. The underlying causes for this trend are not well understood, but are of major concern if those who are young carry this increase with them as they age, which will put additional strain on the healthcare system. Therefore, information regarding occurrence of stroke is important for planning of future strategies in prevention and care.

The absolute number of cases of stroke is expected to increase in the near future because of the growing older population. According to a recent report from Canada, the projected number of people experiencing effects of stroke will increase by between 62% and 79% from 2013 to 2038 [8]. Similar projections have been reported in other regions, including Western Australia and United States, with an overall increase in prevalence over time [9,10]. In Sweden, the majority of all stroke cases (~85%) are ischemic stroke (IS), but trends and current prevalence of IS have not been well quantified in the Swedish population. The present study aimed to investigate age- and sex-specific time trends of the prevalence and absolute number of IS survivors in Sweden from 1995–2010.

## Materials and methods

### Study population

In Sweden, a universal health care system is provided at a low cost to all citizens. Hospitals are required to report the principal and contributory diagnosis to the Inpatient Register (IPR) and the Cause of Death Register. The IPR was established in 1964 and has been operating on a national basis since 1987 with a complete national coverage of all hospital discharge diagnoses, while the Cause of Death Register has been in operation since 1961. For the purpose of this study, data were obtained by linkage with the IPR and Cause of Death Register through the Swedish 10-digit personal identifier.

In the present study, all men and women older than 18 years who were hospitalized at least once during 1995–2010 and survived with a principal discharge diagnosis of IS were included. Patients were stratified according to age group (18–44, 45–54, 55–64, 65–74, 75–84, and ≥85 years) and admission years. All discharge diagnoses were coded according to the International Classification of Disease (ICD) system. The ICD-8 was used from 1968 to 1987, ICD-9 from 1987 to 1996, and ICD-10 from 1996 onwards. Ischemic stroke was coded as ICD-8 and ICD-9 code 434 and 436 and ICD-10 codes I63 and I64.

Administrative data from the Swedish IPR and Cause of Death register was used to estimate the prevalence of IS over time for a specific age and year. The prevalence of IS includes 2 subsets of prevalent cases: 1) incident cases that survive up to an index date and 2) cases that were incident before the start of the registry and survived up to the index date. The second data set was created using the mean incidence rate from the IPR to estimate the annual number of incident cases for each age and sex. All survival estimates before 1987 were adjusted to follow the mortality rate in the general population. The counting method used to accumulate prevalent cases of IS for the datasets was described more formally by Gail et al [11]. The Swedish general population from Human Mortality Database was used as the reference group when calculating the prevalence of IS in the population [12]. Prevalence is reported as patients per 10,000 population.

### Statistical analysis

Prevalence and survival were estimated by SAS software version 9.3 (SAS, Cary, NC, USA) and R program version 2X. Joinpoint regression analysis was performed with Joinpoint Regression Program 4.2.0.2 (Statistical Methodology and Applications Branch, Surveillance Research Program, National Cancer Institute). The prevalence (proportions) was used as dependent variable and year (1995–2010) was used as independent variable, the analysis was stratified by age group. A linear model with minimum four data point between each jointpoint and a maximum of 2 joinpoints were allowed in which the software finds the optimal number of joinpoints. The slopes (S) and intercepts were estimated using least squares and the number and position of the joinpoints were automatically selected using grid search. Within each interval, the APC were calculated as: (exp (Si)-1)*100, and the average APC (AAPC) were calculated from the weighted average (with number of data points within each interval as weight) of slopes. The upper and lower 95% confidence interval (CI) for the APC within each interval was calculated from the estimated variances of the slopes (Vi): (exp(Si+/-t*Vi-1)*100, and the 95% CI of the AAPC was calculated from the pooled variance and average slope.

### Ethics statement

This study was approved by the Regional Ethical Review Board of Gothenburg University (number 540–11). For anonymity, all personal identifiers were removed and replaced with a code in the final data set.

### Results

From 1995–2010, the number of patients with IS aged 18 to ≥85 years in Sweden increased from 129,418 to 148,778. The number of male patients increased from 70,958 to 79,817 and that of female patients increased from 58,460 to 68,961, corresponding to a relative increase of 12.5% and 18.0%, respectively (S1 Table). The largest relative increase was observed in age groups 55–64 years and ≥85 years, by 49% and 28%, respectively, in men and 61% and 47%, respectively, in women (S1B Fig for men and S1C Fig for women). During the same period there was an approximate increase of ~40% in the total population size among those aged 55–64 years and ≥85 years (S2 Fig).

### Overall prevalence

Table 1 shows the prevalence of IS in the total population and Table 2 the joinpoint analysis of trends in prevalence in Sweden by age, sex and year. The overall prevalence of surviving patients who had ever been hospitalized with IS aged 18 to ≥85 years was 189 patients per 10,000 population in 1995, which increased to 202 patients per 10,000 population in 2000 with an APC of 1.6% (95% CI 1.4 to 1.7). The prevalence remained stable until 2006, followed by a decrease (APC: -0.8%, 95% CI -1.0 to -0.6) and with a final prevalence of 199 patients per 10,000 population in 2010.

**Table 1:** Prevalence of patients hospitalized with ischemic stroke in the total population divided by age groups, sex and calendar year from 1995–2010.

Age group (years), Prevalence per 10,000 population	1995	1996	1997	1998	1999	2000	2001	2002	2003	2004	2005	2006	2007	2008	2009	2010
**Overall**																
18–44	6.4	6.6	6.8	6.9	6.9	6.9	7.1	7.1	7.0	7.2	7.3	7.3	7.3	7.5	7.4	7.6
45–54	40.1	41.5	42.8	44.4	45.2	45.5	45.1	45.0	44.8	44.2	43.3	43.4	43.7	43.4	43.2	43.8
55–64	137	139	141	142	143	144	144	144	145	147	149	151	152	152	151	151
65–74	415	420	423	427	433	434	436	435	431	425	422	418	410	404	397	393
75–84	912	909	920	935	945	950	956	961	961	961	961	960	946	940	927	920
85+	1471	1456	1456	1460	1464	1471	1477	1481	1473	1472	1447	1448	1450	1443	1436	1453
Total	189	191	194	198	201	202	204	205	205	205	205	205	203	202	200	199
**Men**																
18–44	6.0	6.0	6.2	6.3	6.3	6.4	6.6	6.4	6.3	6.5	6.6	6.6	6.7	7.0	6.9	7.1
45–54	48.3	49.7	51.7	53.3	54.0	53.9	53.7	53.1	52.8	51.9	50.9	51.4	51.8	51.0	50.6	50.7
55–64	181	183	185	187	186	186	186	185	187	187	190	191	192	193	191	191
65–74	556	561	559	563	572	571	568	567	558	550	545	539	527	521	510	503
75–84	1190	1174	1178	1183	1187	1185	1187	1189	1188	1182	1180	1177	1162	1149	1137	1127
85+	2139	2070	2048	2020	1987	1977	1965	1946	1913	1903	1833	1811	1799	1772	1772	1792
Total	212	212	215	217	219	220	221	222	221	221	221	221	219	218	217	217
**Women**																
18–44	6.9	7.2	7.4	7.5	7.5	7.5	7.7	7.7	7.9	7.9	8.0	8.0	8.0	7.9	8.0	8.1
45–54	32	33	34	35	36	37	36	37	37	36	35	35	35	36	36	37
55–64	94	96	97	98	99	100	101	102	104	106	108	111	112	112	111	110
65–74	295	300	306	310	313	315	319	318	318	312	311	306	301	294	290	287
75–84	722	727	743	762	776	785	794	799	799	803	804	803	789	785	771	764
85+	1177	1185	1195	1213	1233	1246	1259	1271	1272	1272	1266	1275	1281	1282	1270	1284
Total	167	170	175	179	183	185	188	189	189	189	190	190	188	187	184	183

**Table 2:** Jointpoint analysis of trends in prevalence of ischemic stroke among men and women in Sweden from 1995–2010.

Age group (years)	Period 1 Years	APC (95% CI)^a^	Period 2 Years	APC (95% CI) ^a^	Period 3 Years	APC (95% CI) ^a^
**Overall**						
18–44	1995–1998	2.1 (0.9 to 3.4)*	1998–2010	0.7 (0.6 to 0.9)*	-	-
45–54	1995–1999	3.3 (2.7 to 4.0)*	1999–2006	-0.7 (-1.1 to -0.4)*	2006–2010	0.1 (-0.6 to 0.7)
55–64	1995–2004	0.7 (0.5 to 0.8)*	2004–2007	1.3 (-0.4 to 3.0)	2007–2010	-0.4 (-1.3 to 0.4)
65–74	1995–2001	0.9 (0.7 to 1.0)*	2001–2006	-1.0 (-1.2 to -0.7)*	2006–2010	-1.6 (-1.8 to -1.3)*
75–84	1995–2001	1.0 (0.7 to 1.2)*	2001–2006	0.0 (-0.4 to 0.4)	2006–2010	-1.1 (-1.5 to -0.7)*
≥85	1995–2002	0.1 (-0.1 to 0.4)	2002–2010	-0.3 (-0.5 to -0.1)*	-	-
Total	1995–2000	1.6 (1.4 to 1.7)*	2000–2006	0.1 (-0.0 to 0.3)	2006–2010	-0.8 (-1.0 to -0.6)*
**Men**						
18–44	1995–2010	1.0 (0.8 to 1.2)*	-	-	-	-
45–54	1995–1999	2.8 (2.0 to 3.7)*	1999–2010	-0.7 (-0.9 to -0.5)*	-	-
55–64	1995–2010	0.3 (0.3 to 0.4)*	-	-	-	-
65–74	1995–2001	0.5 (0.3 to 0.8)*	2001–2010	-1.4 (-1.5 to -1.3)*	-	-
75–84	1995–2005	0.0 (-0.1 to 0.1)	2005–2010	-1.0 (-1.3 to -0.8)*	-	-
≥85	1995–2010	-1.2 (-1.4 to -1.1)*	-	-	-	-
Total	1995–2000	0.9 (0.7 to 1.1)*	2000–2005	0.0 (-0.2 to 0.2)	2005–2010	-0.4 (-0.6 to -0.3)*
**Women**						
18–44	1995–1997	3.3 (0.4 to 6.3)*	1997–2005	1.0 (0.6 to 1.3)*	2005–2010	0.1 (-0.5 to 0.8)
45–54	1995–2000	3.1 (2.3 to 3.9)*	2000–2007	-0.7 (-1.3 to -0.2)*	2007–2010	1.2 (-0.5 to 2.9)
55–64	1995–2003	1.2 (1.1 to 1.3)*	2003–2007	2.1 (1.6 to 2.6)*	2007–2010	-0.6 (-1.1 to -0.1)*
65–74	1995–2001	1.3 (1.0 to 1.6)*	2001–2005	-0.8 (-1.6 to 0.0)	2005–2010	-1.7 (-2.0 to -1.3)*
75–84	1995–2000	1.9 (1.6 to 2.2)*	2000–2005	0.5 (0.0 to 0.9)	2005–2010	-1.1 (-1.4 to -0.8)*
≥85	1995–2002	1.1 (1.0 to 1.3)*	2002–2010	0.1 (-0.1 to 0.2)	-	-
Total	1995–2000	2.3 (2.2 to 2.5)*	2000–2006	0.3 (0.1 to 0.5)*	2006–2010	-1.1 (-1.3 to -0.8)*

^a^APC = Annual percentage change

*Significantly different from zero at α = 0.05.

In the youngest age group of 18–44 years, the prevalence increased with an APC of 2.1% (95% CI 0.9 to 3.4) from 6.4 in 1995 to 6.9 patients per 10,000 population in 1998. The prevalence continued to increase but at a slower pace with an APC of 0.7% (95% CI 0.6 to 0.9) throughout the study and with a final prevalence of 7.6 patients per 10,000 population in 2010. An increase in overall prevalence was observed among those aged 45–54 years with an APC of 3.3% (95% CI 2.7 to 4.0) during 1995–1999 and by 0.7% (95% CI 0.5 to 0.8) among 55–64 years during 1995–2004. For those aged 45–54 years the prevalence decreased (APC: -0.7%, 95% CI -1.1 to -0.4) until 2006, after which it remained stable throughout the study with a final prevalence of 43.8 patients per 10,000 population in 2010. Among 55–64 years, there were no significant changes in prevalence after 2004 and with a final prevalence of 151 patients per 10,000 population in 2010. For those aged 65–74 years, the overall prevalence of IS increased with an APC of 0.9% (95% CI 0.7 to 1.0) from 415 in 1995 to 436 patients per 10,000 population in 2001. This was then followed by a decrease (APC: -1.0%, 95% CI -1.2 to -0.7) until 2006 which continued throughout the study but at higher rate (APC: -1.6%, 95% CI -1.8 to -1.3) and with a final prevalence of 393 patients per 10,000 population in 2010. Among persons aged 75–84 years, the prevalence increased with an APC of 1.0% (95% CI 0.7 to 1.2) from 912 in 1995 to 956 patients per 10,000 population in 2001. The prevalence remained stable until 2006 which was then followed by a decrease (APC: -1.1%, 95% CI -1.5 to -0.7) to 920 patients per 10,000 population in 2010. In the oldest age group ≥85 years, the overall prevalence was estimated as 1471 patients per 10,000 population in 1995 which remained stable until 2002. Afterwards there was a slight decline in prevalence (APC: -0.3%, 95% CI -0.5 to -0.1) throughout the study to 1453 patients per 10,000 population in 2010.

### Trends in age- and sex-specific prevalence

**Fig 1:**
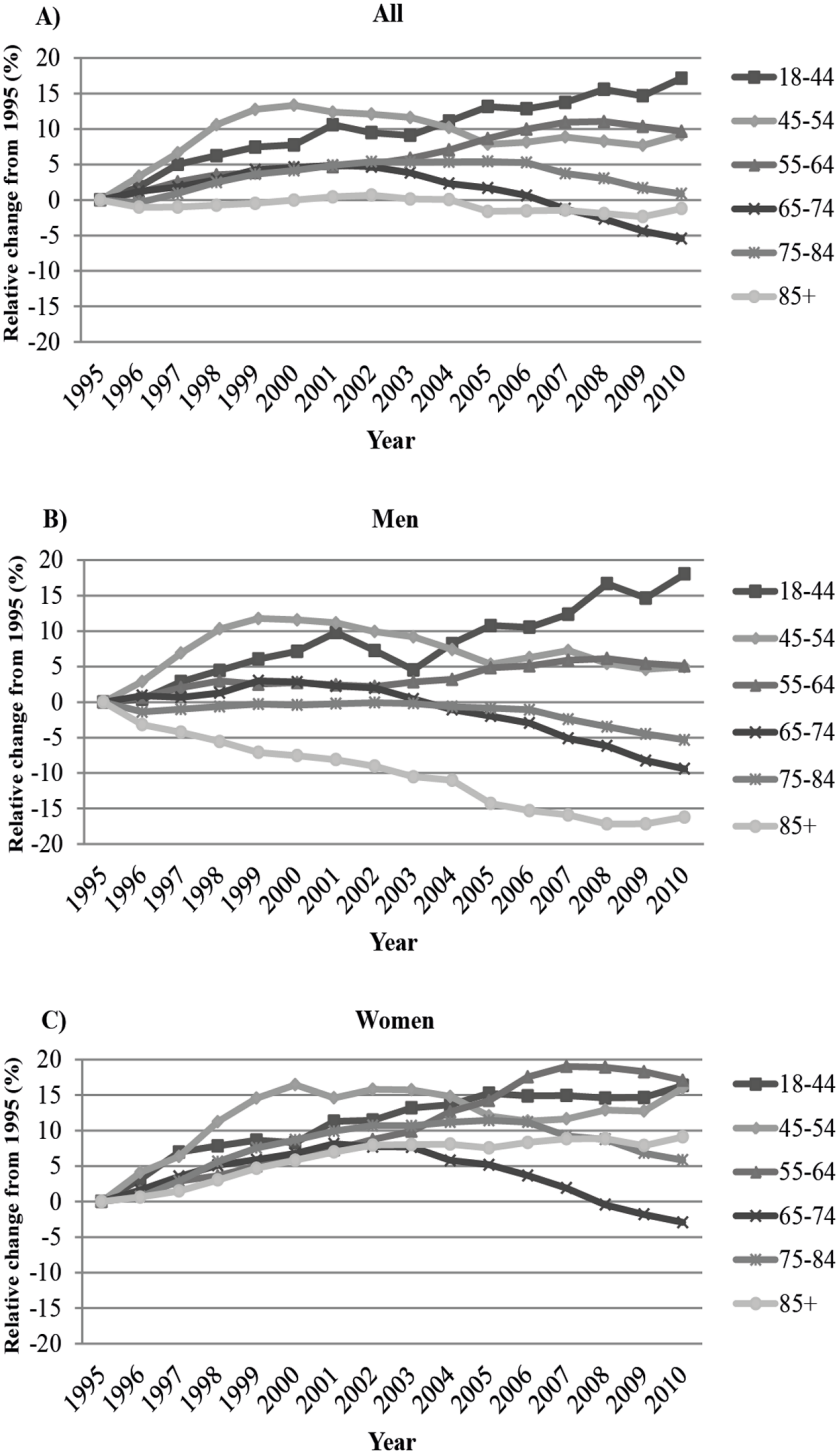
Relative change in the prevalence of IS divided by age groups in A) all patients, B) in men, and C) in women.

The overall prevalence of IS in men aged 18 to ≥85 years increased with an APC of 0.9% (95% CI 0.7 to 1.1) from 212 in 1995 to 220 patients per 10,000 population 2000 and then remained stable until 2005, followed by a decrease (APC: -0.4%, 95% CI -0.6 to -0.3) to 217 patients per 10,000 population in 2010 (Tables 1 and 2). Among women the overall prevalence increased with an APC of 2.3% (95% CI 2.2 to 2.5) from 167 in 1995 to185 patients per 10,000 population in 2000. The prevalence rates continued to increase (APC: 0.3%, 95% CI 0.1 to 0.5) but at a slower pace until 2006 and with a prevalence of 190 patients per 10,000 population, then declining with an APC of -1.1% (95% CI -1.3 to -0.8) to 183 patients per 10,000 population in 2010. Trends in the overall prevalence of IS differed markedly by age and sex. In men, the prevalence in those aged 18–44 years increased slightly but continuously with an APC of 1.0% (95% CI 0.8 to 1.2) throughout the study from 6.0 in 1995 to 7.1 patients per 10,000 population in 2010, a relative increase of 18.3% (Table 1 and Fig 1B). The prevalence among those aged 45–54 years increased during the first years with an APC of 2.8%, (95% CI 2.0 to 3.7) until 1999, followed by a slight decrease (APC -0.7, 95% CI -0.9 to -0.5) throughout the study and with a final prevalence of 50.7 patients per 10,000 population in 2010.

For women, a similar pattern was observed among those aged 18–44 years. Prevalence increased with an APC of 3.3% (95% CI 0.4 to 6.3) from 6.9 in 1995 to 7.4 patients per 10,000 population in 1997. The rates continued to increase from 1997–2005 but at a slower pace (APC: 1.0%, 95% CI 0.6 to 1.3) after which the rates remained stable throughout the study with a final prevalence of 8.1 patients per 10,000 population in 2010, and with a relative increase of 17.4% (Fig 1C). During 1995–2000 the prevalence among women aged 45–54 years increased with an APC of 3.1% (95% CI 2.3 to 3.9) from 32 to 37 patients per 10,000 population, followed by a slight decrease until 2007 (APC: -0.7%, 95% CI -1.3 to -0.2) and then remained stable throughout the study with a final prevalence of 37 patients per 10,000 population in 2010. The prevalence of IS among men aged 55–64 years increased throughout the study with an APC of 0.3% (95% CI 0.3 to 0.4) from 181 in 1995 to 191 patients per 10,00 population in 2010. Among women aged 55–64 years there was an initial increase (APC: 1.2%, 95% CI 1.1 to 1.3) in prevalence from 94 in 1995 to 104 patients per 10,000 population in 2003. This was then followed by a steeper increase (APC: 2.1%, 95% CI 1.6 to 2.6), reaching a peak in 2007, after which there was a minor decline (APC:-0.6%, 95% CI -1.1 to -0.1) and with a final prevalence of 110 patients per 10,000 population in 2010. The prevalence among those aged 65–74 years increased with an APC of 0.5% (95% CI 0.3 to 0.8) in men from 556 in 1995 to 568 patients per 10,000 population in 2001. Corresponding results for women was 295 to 319 patients per 10,000 population and with an APC of 1.3% (95% CI 1.0 to 1.6) during 1995–2001. The rates then decreased in men (APC: -1.4%, 95% CI -1.5 to -1.3) throughout the study, but not in women where it remained stable until 2005, followed by a decline (APC: -1.7%, 95% CI -2.0 to -1.3) and with a final prevalence of 503 and 287 patients per 10,000 population, respectively, in 2010. From 1995–2000 the prevalence rate of IS among those aged 75–84 years increased in women (APC: 1.9%, 95% CI 1.6 to 2.2) from 722 to 799 patients per 10,000 population while it remained stable in men. The rates in women continued to increase but at a slower pace until 2005 (APC: 0.5%, 95% CI 0.0 to 0.9). After 2005, the rates decreased with an APC of -1.0% (95% CI -1.3 to -0.8) in men and by -1.1% (95% CI -1.4 to -0.8) in women throughout the study, with a final prevalence of 1127 and 764 patients per 10,000 population, respectively, in 2010. In the oldest age group of ≥85 years, the prevalence of IS decreased with an APC of -1.2% (95% CI -1.4 to -1.1) throughout the study in men from 2139 in 1995 to 1792 patients per 10,000 population in 2010. Among women the prevalence increased with an APC of 1.1% (95% CI 1.0 to 1.3) from 1177 in 1995 to 1271 patients per 10,000 population in 2002 after that the rates of IS remained stable, with a final prevalence of 1284 patients per 10,000 population, in 2010.

## Discussion

Our study shows that the overall number of patients who were hospitalized with IS increased by 12.5% in men and 18.0% in women from 1995–2010. An overall increase in prevalence of IS was observed during most of the mid to late 1990s, but leveled off after 2000 and remained stable until 2006, followed by a slight decline (APC: -0.8%, 95% CI -1.0 to -0.6) and with a final prevalence of 199 patients per 10,000 population in 2010 (Tables 1 and 2). Age-specific trends in prevalence markedly differed. In the youngest age group of 18–44 years, the prevalence of IS increased throughout the study period. However, in those aged 45–54 years the prevalence increased from 1995–1999, followed by a slight decline (APC: -0.7%, 95% CI -1.1 to -0.4) until 2006 and then remained stable at 43.8 patients per 10,000 population in 2010. In the oldest age group of ≥85 years the overall prevalence of IS remained stable during the first years of the study, after 2002 there was a slight decline (APC: -0.3%, 95% CI -0.5 to -0.1) until 2010 with a final estimate of 1453 patients per 10,000 population.

Most studies on stroke prevalence have only considered overall stroke while we estimated IS only. In an Italian study, the overall prevalence of stroke in persons aged ≥15 years was estimated as 1.47% in 2004, but this was based on comparatively few cases [13]. In England, Scotland, and Wales, the prevalence was 1.7% in 2006/2007, while in Australia, the estimate prevalence in 2009 was 1.9% in men and 1.3% in women [14,15]. Canada reported a prevalence of stroke of 1.14% in 2009/2010, and in United States, stroke prevalence remained stable at 2.6% from 2006–2010 [8,16]. Previous studies were either based on specific regions, self-reported, a national survey, or conducted only for a short period of time [13,15,16]. We aimed to investigate trends in the prevalence of IS for a whole nation over time based on national hospital records, based on the net effect of the incidence of IS and mortality for each year. This provides the absolute number of patients with IS and enabled us to estimate trends in prevalence of IS. We found that the overall prevalence of IS in Sweden increased during the mid to late 1990s until the beginning of the 2000s, and then remained stable until 2006 after which it declined slightly until 2010 (199 per 10,000 population), but with varying trends between age groups (Table 1). Decline in overall prevalence of IS might to some extent be explained by improvements in treatments and changes in cardiovascular risk factors over time e.g. lower blood pressure levels and smoking rates [17–20]. This might be explained by dietary changes and better antihypertensive treatment in the general population over time but also due to intervention campaigns and implemented smoking bans in public spaces. Additionally, decreases in cholesterol levels have been observed in Sweden for the past years which explained most of the decline in coronary heart disease mortality in Sweden [20]. Notably, the overall prevalence rate in the youngest age groups steadily increased throughout the study (Table 2). A possible explanation for the increase in prevalence could be due to better diagnostic methods over time such as the use of magnetic resonance imaging. However, this would not have affected only young people in whom we observed the most marked rise in prevalence of IS. Computed tomography scan (CT) has been widely used during the study period to distinguish between ischemic and hemorrhagic stroke. According to a recent study from the Swedish Stroke Register (Riks-Stroke) a majority of stroke patients are examined with CT (>98%) [21]. The overall prevalence of IS was slightly higher in men than in women in all age groups. Similar findings were reported in Western Australia and Canada, but not in the USA, which reported a slightly higher proportion of women in people aged <60 years [8,15,16]. Our results of an increasing prevalence of IS in the younger age groups are in accordance with previous findings of an increasing incidence trend of IS in younger adults [22–24]. The reasons for this are not known, but could potentially be due to increasing sedentary lifestyle and obesity but also to demographic changes in population [25, 26]. Generally, the greatest increase in prevalence of IS in men and women occurred during the mid to late 1990s, followed by a stabilized or declining rate until the end of the study period. Although the prevalence of IS in some age groups remained stable or declined for a few years, the absolute number of patients increased over time, likely due to demographic changes with more people surviving into old age but also because of improved primary and secondary treatments in stroke (S2 Fig). We have demonstrated improved survival after ischemic stroke in IS patients aged 55 and younger, which likely is applicable to older patients as well [3]. The largest proportional increase was observed among those aged 55–64 years and ≥85 years because of the growing numbers of people in the two age groups in the general population. Additionally, recent advances and developments in the healthcare system have led to an overall improvement in survival with improvements in medical treatment and secondary prevention (e.g., increased use of anticoagulation treatment among older people after a stroke) [27–30]. Because stroke is strongly correlated with age, quantifying the numbers of stroke is an important task for future healthcare planning.

### Strengths and limitations

There are limitations and strengths to our study. To investigate trends in prevalence over time, large populations are often needed to identify a sufficient amount of people with IS. Therefore, the main strength of our study is the use of data from an entire nation. Because of our unique personal identification numbers, all people could be followed throughout the whole study period with virtually no loss to follow-up. This enabled study of age-specific trends in prevalence over a long time in men and women separately. Limitations include the lack of externally validated stroke diagnoses over time. However, previous studies on the use of principal diagnosis from the Swedish IPR have shown a high validity of many cardiovascular diagnoses, including stroke, especially after 2000 [31, 32]. A limitation in the present study is the lack of information on diagnostic methods because improved medical imaging modalities over time could increase detection rates of IS. However, CT scan has been widely used during the study period and is still the most common method in the evaluation of stroke and accordingly changes in detection rates are unlikely to explain the trends that we observed, or the variation between age groups [21].

## Conclusions

In conclusion, the overall prevalence of hospitalized patients with IS has increased for the past years in Sweden but at different rates over time. In most age groups, the prevalence of IS increased during the mid to late 1990s and then declined or remained stable, except in the youngest age groups where the prevalence increased throughout the study. Additionally, the absolute number of patients with IS has increased in most age groups from 1995–2010, especially in those aged 55–64 years and ≥85 years, which could mainly be explained by demographic factors. If this trend continues into the future, it will cause additional strain to the healthcare system.

## Supporting information

S1 Fig Relative change in number of patients hospitalized with IS from 1995–2010 by age group in A) all patients, B) in men and C) women only. (TIF)

S2 Fig Relative demographic change in population size from 1995–2010 in the Swedish population by age groups. (TIF)

S1 Table Number of patients hospitalized with an ischemic stroke divided by age groups, sex and calendar year from 1995–2010. (DOCX)
