# Supplementary figures and images for: Long-term trends in the prevalence of patients hospitalized with ischemic stroke from 1995 to 2010 in Sweden
Source: PLoS One. 2017 Jun 16;12(6):e0179658. doi: 10.1371/journal.pone.0179658 (PMC5473590; doi:10.1371/journal.pone.0179658)

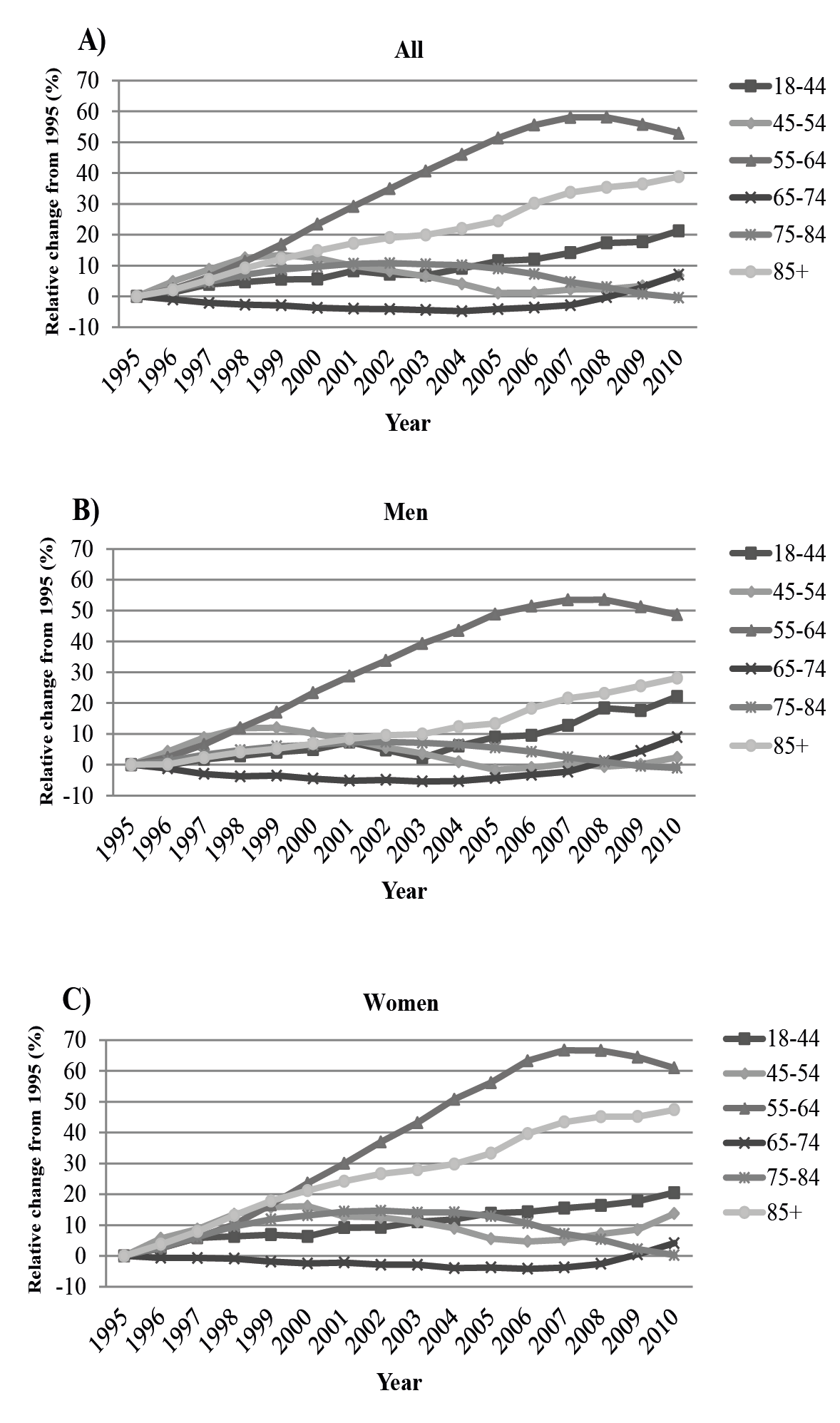

Supplement: S1 Fig — (TIF) [file pone.0179658.s001.tif]

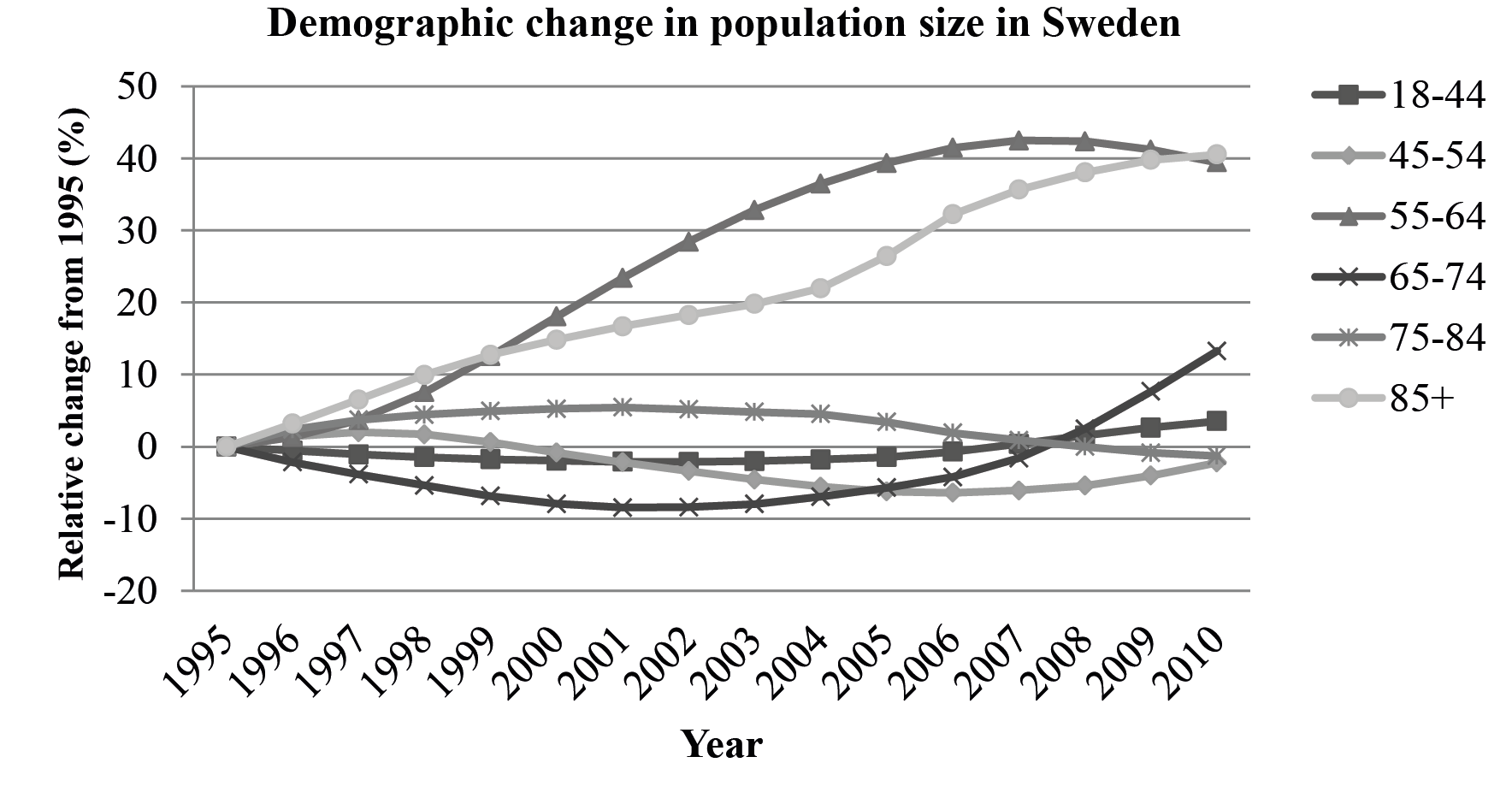

Supplement: S2 Fig — (TIF) [file pone.0179658.s002.tif]
