# Supplementary material for: Long-term trends in the prevalence of patients hospitalized with ischemic stroke from 1995 to 2010 in Sweden
Source: PLoS One. 2017 Jun 16;12(6):e0179658. doi: 10.1371/journal.pone.0179658 (PMC5473590; doi:10.1371/journal.pone.0179658)
Supplement: S1 Table — (DOCX) [file pone.0179658.s003.docx]

| **S1 Table 1. Number of patients hospitalized with an ischemic stroke divided by age groups, sex and calendar year from 1995-2010.** | | | | | | | | | | | | | | | | |
| --- | --- | --- | --- | --- | --- | --- | --- | --- | --- | --- | --- | --- | --- | --- | --- | --- |
|  |  |  |  |  |  |  |  |  |  |  |  |  |  |  |  |  |
|  | **1995** | **1996** | **1997** | **1998** | **1999** | **2000** | **2001** | **2002** | **2003** | **2004** | **2005** | **2006** | **2007** | **2008** | **2009** | **2010** |
| **Overall** |  |  |  |  |  |  |  |  |  |  |  |  |  |  |  |  |
| 18-44 | 2077 | 2103 | 2158 | 2174 | 2192 | 2194 | 2249 | 2225 | 2220 | 2266 | 2316 | 2327 | 2371 | 2437 | 2444 | 2519 |
| 45-54 | 4992 | 5233 | 5429 | 5615 | 5664 | 5612 | 5488 | 5405 | 5317 | 5194 | 5048 | 5053 | 5104 | 5111 | 5160 | 5330 |
| 55-64 | 11746 | 12027 | 12490 | 13079 | 13730 | 14499 | 15175 | 15846 | 16523 | 17160 | 17786 | 18274 | 18570 | 18572 | 18302 | 17965 |
| 65-74 | 33554 | 33222 | 32849 | 32656 | 32582 | 32305 | 32209 | 32165 | 32064 | 31944 | 32170 | 32342 | 32599 | 33461 | 34541 | 35930 |
| 75-84 | 50777 | 51789 | 53147 | 54342 | 55209 | 55659 | 56160 | 56269 | 56066 | 55916 | 55347 | 54463 | 53133 | 52306 | 51182 | 50559 |
| 85+ | 26272 | 26832 | 27701 | 28674 | 29477 | 30162 | 30794 | 31276 | 31512 | 32060 | 32688 | 34205 | 35128 | 35567 | 35858 | 36475 |
| Total | 129418 | 131206 | 133774 | 136540 | 138854 | 140431 | 142075 | 143186 | 143702 | 144540 | 145355 | 146664 | 146905 | 147454 | 147487 | 148778 |
| **Men** |  |  |  |  |  |  |  |  |  |  |  |  |  |  |  |  |
| 18-44 | 985 | 983 | 1002 | 1013 | 1025 | 1033 | 1057 | 1032 | 1007 | 1045 | 1073 | 1079 | 1110 | 1166 | 1158 | 1203 |
| 45-54 | 3058 | 3189 | 3327 | 3420 | 3425 | 3366 | 3307 | 3229 | 3169 | 3087 | 3006 | 3028 | 3068 | 3039 | 3061 | 3131 |
| 55-64 | 7659 | 7840 | 8162 | 8573 | 8966 | 9448 | 9861 | 10247 | 10669 | 10996 | 11402 | 11599 | 11755 | 11762 | 11579 | 11386 |
| 65-74 | 20698 | 20448 | 20078 | 19912 | 19965 | 19765 | 19628 | 19677 | 19574 | 19599 | 19797 | 20022 | 20225 | 20937 | 21617 | 22541 |
| 75-84 | 26891 | 27222 | 27765 | 28165 | 28490 | 28634 | 28838 | 28875 | 28808 | 28651 | 28377 | 28031 | 27536 | 27142 | 26773 | 26610 |
| 85+ | 11667 | 11677 | 11934 | 12145 | 12273 | 12460 | 12648 | 12781 | 12827 | 13104 | 13222 | 13807 | 14182 | 14368 | 14652 | 14946 |
| Total | 70958 | 71359 | 72268 | 73228 | 74144 | 74706 | 75339 | 75841 | 76054 | 76482 | 76877 | 77566 | 77876 | 78414 | 78840 | 79817 |
| **Women** |  |  |  |  |  |  |  |  |  |  |  |  |  |  |  |  |
| 18-44 | 1092 | 1120 | 1156 | 1161 | 1167 | 1161 | 1192 | 1193 | 1213 | 1221 | 1243 | 1248 | 1261 | 1271 | 1286 | 1316 |
| 45-54 | 1934 | 2044 | 2102 | 2195 | 2239 | 2246 | 2181 | 2176 | 2148 | 2107 | 2042 | 2025 | 2036 | 2072 | 2099 | 2199 |
| 55-64 | 4087 | 4187 | 4328 | 4506 | 4764 | 5051 | 5314 | 5599 | 5854 | 6164 | 6384 | 6675 | 6815 | 6810 | 6723 | 6579 |
| 65-74 | 12856 | 12774 | 12771 | 12744 | 12617 | 12540 | 12581 | 12488 | 12490 | 12345 | 12373 | 12320 | 12374 | 12524 | 12924 | 13389 |
| 75-84 | 23886 | 24567 | 25382 | 26177 | 26719 | 27025 | 27322 | 27394 | 27258 | 27265 | 26970 | 26432 | 25597 | 25164 | 24409 | 23949 |
| 85+ | 14605 | 15155 | 15767 | 16529 | 17204 | 17702 | 18146 | 18495 | 18685 | 18956 | 19466 | 20398 | 20946 | 21199 | 21206 | 21529 |
| Total | 58460 | 59847 | 61506 | 63312 | 64710 | 65725 | 66736 | 67345 | 67648 | 68058 | 68478 | 69098 | 69029 | 69040 | 68647 | 68961 |
